# A new ensemble learning method stratified sampling blending optimizes conventional blending and improves prediction performance

**DOI:** 10.1093/bioadv/vbaf002

**Published:** 2025-02-22

**Authors:** Na Miao, Mengke Yang, Pingping Han, Jiakun Qiao, Zhaoxuan Che, Fangjun Xu, Xiangyu Dai, Mengjin Zhu

**Affiliations:** Key Lab of Agricultural Animal Genetics, Breeding, and Reproduction of Ministry of Education, Huazhong Agricultural University, Wuhan 430070, China; 华中农业大学农业动物遗传育种与繁殖教育部重点实验室，武汉 430070; Key Lab of Agricultural Animal Genetics, Breeding, and Reproduction of Ministry of Education, Huazhong Agricultural University, Wuhan 430070, China; 华中农业大学农业动物遗传育种与繁殖教育部重点实验室，武汉 430070; Key Lab of Agricultural Animal Genetics, Breeding, and Reproduction of Ministry of Education, Huazhong Agricultural University, Wuhan 430070, China; 华中农业大学农业动物遗传育种与繁殖教育部重点实验室，武汉 430070; Key Lab of Agricultural Animal Genetics, Breeding, and Reproduction of Ministry of Education, Huazhong Agricultural University, Wuhan 430070, China; 华中农业大学农业动物遗传育种与繁殖教育部重点实验室，武汉 430070; Key Lab of Agricultural Animal Genetics, Breeding, and Reproduction of Ministry of Education, Huazhong Agricultural University, Wuhan 430070, China; 华中农业大学农业动物遗传育种与繁殖教育部重点实验室，武汉 430070; Key Lab of Agricultural Animal Genetics, Breeding, and Reproduction of Ministry of Education, Huazhong Agricultural University, Wuhan 430070, China; 华中农业大学农业动物遗传育种与繁殖教育部重点实验室，武汉 430070; Key Lab of Agricultural Animal Genetics, Breeding, and Reproduction of Ministry of Education, Huazhong Agricultural University, Wuhan 430070, China; 华中农业大学农业动物遗传育种与繁殖教育部重点实验室，武汉 430070; Key Lab of Agricultural Animal Genetics, Breeding, and Reproduction of Ministry of Education, Huazhong Agricultural University, Wuhan 430070, China; The Cooperative Innovation Center for Sustainable Pig Production, Huazhong Agricultural University, Wuhan 430070, China; 华中农业大学农业动物遗传育种与繁殖教育部重点实验室，武汉 430070; 华中农业大学生猪健康养殖协同创新中心，武汉 430070

## Abstract

**Motivation:**

Ensemble learning, as a powerful machine learning method, improves overall prediction performance by combining the prediction results of multiple base models. Blending, as a popular ensemble learning method, can train multiple base models, input the resulting prediction results to further train meta model and obtain final prediction results. However, conventional blending divides the training set by simple random sampling, which causes bias and large variance, thus affecting the stability and accuracy of prediction performance. In this study, we propose a new algorithm of stratified sampling blending (ssBlending), which addresses the algorithm instability of conventional blending caused by the random partition of the training set, further improving the prediction accuracy.

**Results:**

We used multiple genotype data sets from different species including animal (pig), plant (loblolly pine), and microorganism (yeast) to test the prediction performance of ssBlending. The across-species multi-dataset verification results reveal that ssBlending is superior to conventional blending in terms of prediction accuracy and stability. In addition, we optimized the training set sampling rate (BestH) to facilitate the practical application of the ssBlending algorithm. In summary, this study proposes a completely new algorithm combing stratification strategy with the conventional blending, which provides more options for ensemble learning in various fields.

**Availability and implementation:**

https://figshare.com/s/23122a18dc8a35f12ff6.

## 1 Introduction

In the informationized age, the growth rate of data volume far exceeds human processing capabilities, and the emergence of machine learning is expected to address this problem (Samuel 1959). Machine learning is a computing program that automatically mines the hidden feature information in data. Due to its excellent performance, machine learning has received the increasing attention. Machine learning, as one of the core areas of artificial intelligence (Michalski *et al.* 2013; Helm *et al.* 2020), can accomplish autonomous prediction, classification (Lau and Wu 2003, Li and Zhou 2007, Huang *et al.* 2008, Ben-Haim and Tom-Tov 2010), and some challenging tasks by training and analyzing big data (Papadimitriou and Sun 2008, Havens *et al.* 2012, Zhang *et al.* 2012). Machine learning algorithms can be divided into supervised learning, unsupervised learning, and reinforcement learning. Common machine learning algorithms mainly include support vector machines, decision trees, linear regression, naive Bayes, random forest, elastic net regression, and so on (Mahesh 2020), which have been widely used in various fields (Jordan and Mitchell 2015, Sarker 2021). However, the prediction accuracy of single machine learning method is far from satisfactory. The high performance of machine learning relies on appropriate parameter settings, but the complexity of parameter tuning greatly hinders its application in practical projects. With the increasing scale and complexity of data, it is impractical to employ a single machine learning model in all scenarios (Töscher *et al.* 2009).

Ensemble learning combining multiple learners can be categorized into homogeneous ensemble learning and heterogeneous ensemble learning, based on the composition of individual learners (Zhou 2012). The ensemble learning in which individual learners are trained by the same algorithm is known as homogeneous ensemble learning, while that containing multiple individual learners which are trained by different algorithms is known as heterogeneous ensemble learning. Ensemble learning has been widely applied in various fields such as financial field for stock price prediction (Khan *et al.* 2022, Hao *et al.* 2023), medical field for risk prediction of prevalent diseases (Huang *et al.* 2020, Nilashi *et al.* 2022, Rezaei *et al.* 2023), and so on (Dong *et al.* 2020, Dai *et al.* 2023, Xu *et al.* 2023, Xu *et al.* 2024a, 2024b). Common ensemble learning methods include bagging (Breiman 1996), boosting (Freund and Schapire 1996), stacking (Wolpert 1992), and blending (Töscher *et al.* 2009).

Blending implements a direct random split of the training set into a holdout set and a validation set, based on a predetermined holdout rate. Blending requires training the base learners and the meta learners only once, and thus it significantly reduces computation time in practical applications, compared to stacking. However, the prediction accuracy and stability of blending is compromised due to its randomness of training set partitioning. To overcome this limitation, the study proposes a new algorithm, namely, stratified sampling blending (ssBlending), which combines the stratified sampling strategy with blending. Compared to conventional blending, the ssBlending improves the prediction accuracy, meanwhile maintaining its running speed and stability, thus addressing the computational challenges associated with high-dimensional data.

## 2 Methods

### 2.1 Description of datasets

The data used in this study include genotype data for genomic prediction and phenotypic data. The missing phenotypes were excluded, and genotype quality control was conducted using the PLINK software (Purcell *et al.* 2007). Subsequently, dimensionality reduction of the genotype data was performed utilizing the spls package in R (Chung *et al.* 2012). Individuals with missing phenotypes for each trait are deleted, and individual number for six traits of different species across different datasets are summarized in Table 1.

**Table 1.** Number of individuals across different datasets for six traits of pig, loblolly pine, and yeast.

Pig		Loblolly pine		Yeast	
trait	N_ID	trait	N_ID	trait	N_ID
ADG	4046	RT	925	CuSO4	4276
BF	4046	ABA	861	Diamide	4309
BH	3892	THLC	861	BER	4310
OTW	4052	CWAB	861	Formamide	4268
LEA	3866	TSH	861	IAA	4286
LP	3866	C5C6	910	Lactose	3816

#### 2.1.1 Real datasets

The real dataset used in the study contains the data of pig, loblolly pine and yeast.

The pig dataset used in the study contains the data of average daily weight gain (ADG), backfat thickness (BF), body height (BH), off test weight (OTW), loin eye area (LEA), and lean percentage (LP). This dataset has 4555 samples, with 47 258 SNPs.

The loblolly pine data of root number (RT), average branch angle (ABA), total height to the base of the live crown (THLC), crown width across the plant beds (CWAB), total stem height (TSH), and 5- and 6-carbon sugar content (C5C6). This dataset has 926 samples, with 2788 SNPs (Resende Jr *et al.* 2012).

The genotype data of yeast contains 4390 samples, with 28 220 SNPs per sample. Six traits are investigated, namely, the contents of copper sulfate (CuSO4), diamide, berbamine (BER), formamide, indol acetic acid (IAA), and lactose (Bloom *et al.* 2015).

#### 2.1.2 Simulated datasets

The genotype information is randomly extracted from the real pig gene data to obtain the simulated genotype dataset. Phenotypes corresponding to heritability values of 0.2, 0.5, and 0.8 are generated using the simulation phenotype method.

In the data simulation, we set three gradient quantitative trait nucleotide (QTN) numbers 200, 2000, and 5000 for different heritabilities (h2 = 0.2, 0.5, and 0.8) to represent the effect sizes affecting the phenotypic values of the simulated traits. In the simulation process, the QTN effect follows a normal distribution. The dataset required by the study is then simulated based on the chosen heritability value. The phenotypic simulation involves the additive effect value and the residual effect value. The sum of the two effect values is defined as the final simulated phenotypic value of the individual (Tang and Liu 2019). The simulated phenotype value is calculated according to the following formula:


(1) y=Xβ+e

where y represents a phenotype vector with a length of n × 1; X is a genotype matrix of n × m (n, number of simulated individuals; m, number of extracted QTNs); β is a QTN effect vector with a length of n × 1; and e indicates a random residual vector with a length of n × 1.

Finally, we obtained nine simulated datasets with different genetic architectures with each dataset containing 3000 samples and 20 000 SNPs.

### 2.2 Description of methods

#### 2.2.1 Blending

The blending algorithm consists of two layers of learners. The first layer framework is composed of three models as the basic learners, including support vector regression model (Chang and Lin 2011) implemented by the svm function in the e1071 R package, regression tree model (Grubinger *et al.* 2014) by the evtree function in the evtree R package, and elastic net regression model (Durbin and Willshaw 1987) by the glmnet function in the glmnet R package. In the second layer framework, a linear model is used as the meta learner implemented by the lm function in the R software.

#### 2.2.2 ssBlending

The ssBlending algorithm is an evolution of the blending algorithm. The ssBlending also follows a two-layer framework and employs meta learning to generate final predicted values. The conventional blending algorithm randomly selects a holdout set, and this randomness tends to result in strong instability. To address this issue, we propose applying the stratified sampling strategy to the holdout set partition in blending.

**Figure 1.**
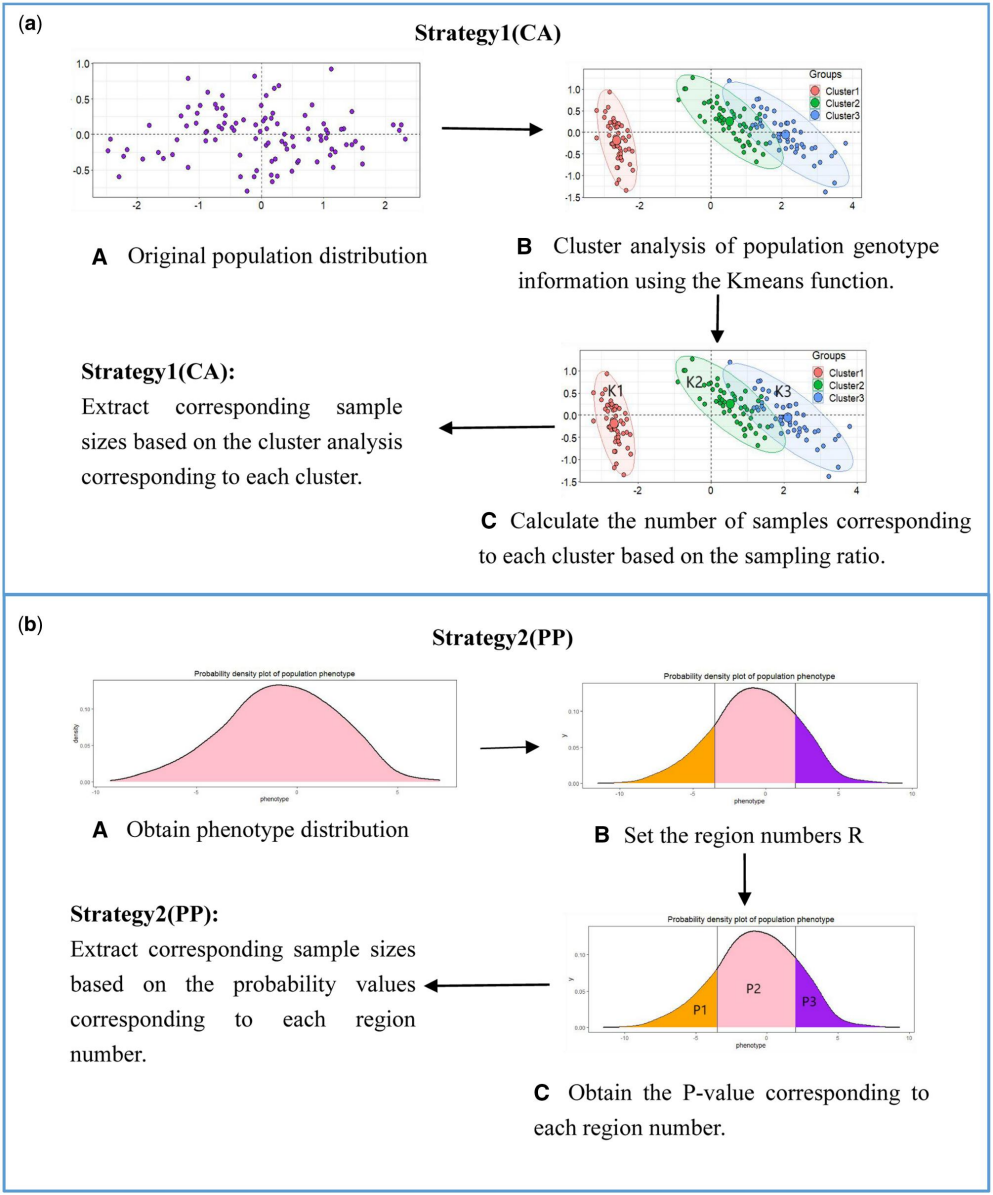
Schematic diagram of two stratification strategies. (a) Cluster analysis strategy (CA) of ssBlending. The genotype information of the population is divided into N categories, and then sampled from N categories. (b) Penotype probability (PP) stratification strategy of ssBlending. The probability density curve of each phenotype data is plotted. The phenotype is divided into several intervals of equal distance, and the probability of different phenotype intervals is calculated using Riemann integral, and finally stratified sampling is performed according to the desired probability. The proposed ssBlending algorithm uses two stratification strategies to reduce the risk of selecting special samples due to the randomness of conventional Blending in dividing training set, and the samples obtained by the stratification strategy can better represent their original characteristics.

In our study, we combine the blending with the cluster analysis stratification strategy (CA) and the phenotype probability stratification strategy (PP), respectively. Figure 1 presents the combination of these approaches. In CA strategy, we adopt the KMeans clustering algorithm implemented by the KMeans function in the R software for clustering purposes.

**Figure 2.**
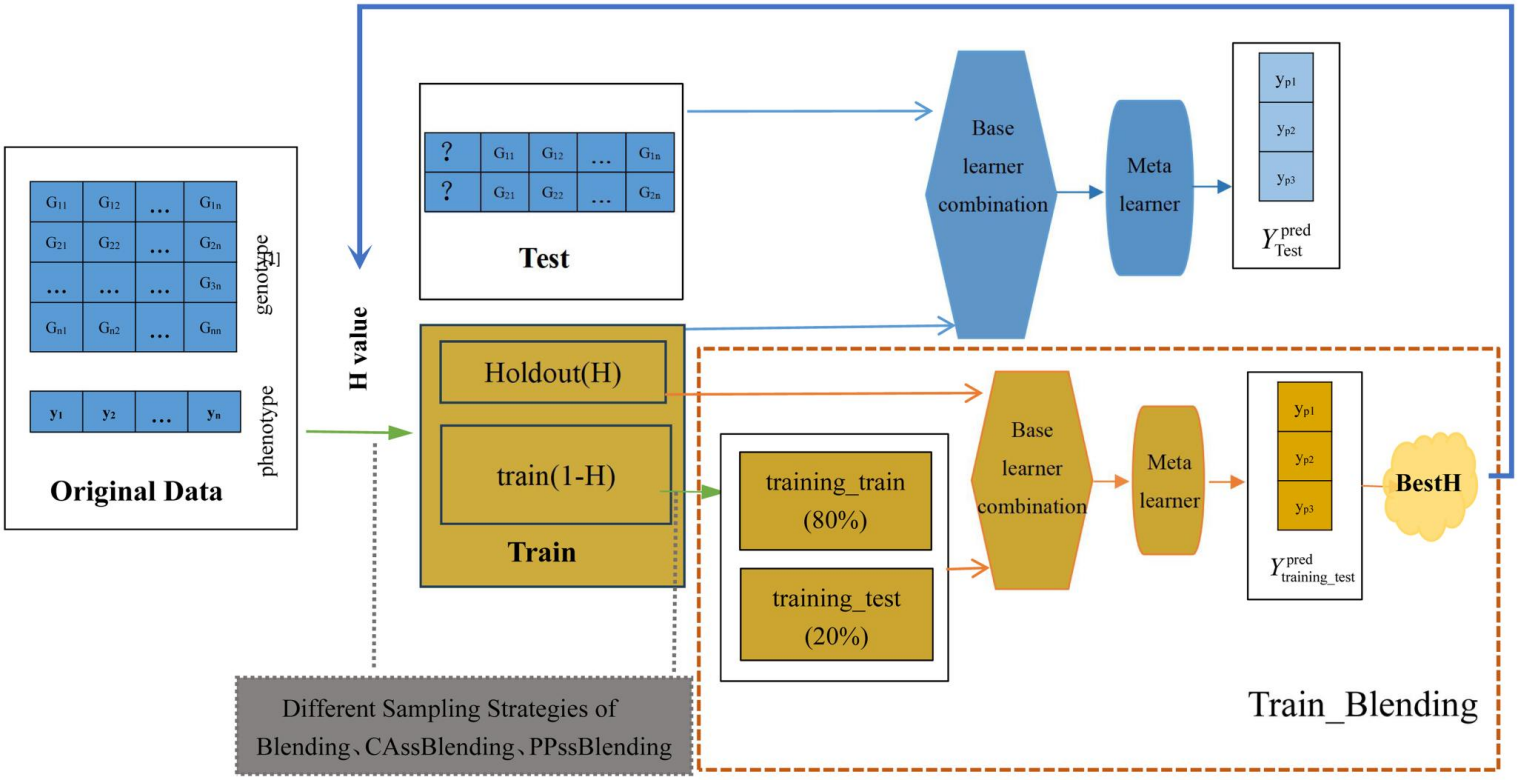
The flowchart of our study. The main process is to acquire the Train_Blending for BestH in the Train set. And then use BestH for the Blending and ssBlending in the original data.

We combine two stratification strategies (CA and PP) with blending for partitioning data, and define them as CAssBlending and PPssBlending, respectively. Subsequently, using Blending, CAssBlending, and PPssBlending, respectively, the phenotype values are predicted on the Test set, and the prediction accuracy of the three Blending methods is evaluated by investigating the Pearson correlation coefficient between the predicted value and the observed value. Figure 2 shows the flowchart of ssBlending.

## 3 Results

### 3.1 Optimization of sampling rate of holdout set

To improve the prediction accuracy of the model, we optimize parameters in model training, since model prediction accuracy is greatly affected by different parameters. For the same computer, the same server, the same computing resources, we set different sampling rates (H = 0.1, 0.15, …, 0.6) in the Blending and ssBlending implementation. We set the number of layers as 8 for both stratification strategies (CA and PP). In addition, we calculate the prediction accuracy on training_test set at different gradient H values. By altering parameter H values we obtain different prediction accuracy values, and the H value corresponding to the highest prediction accuracy is automatically set as BestH. This optimization of H value is conducive to improving prediction accuracy.

### 3.2 Prediction performance of different methods on simulations

**Figure 3.**
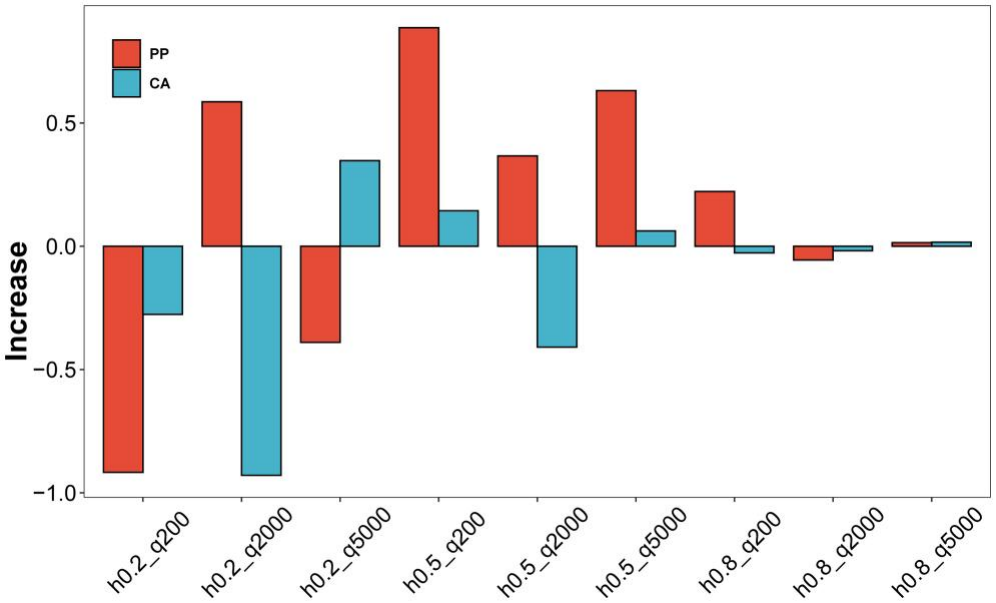
Increase in prediction accuracy of ssBlending, compared with Blending on pig simulated set.

Table 2 shows the prediction performance of the three methods on simulated sets at different heritability values. We found that in most cases, at least one ssBlending strategy (CA and/or PP) outperformed Blending, with an increase in prediction accuracy of 0.1439%–0.8866% (Fig. 3). In the h0.5_q200 dataset, the prediction accuracy of Blending was only 0.5669, whereas that of PPssBlending and CAssBlending reached 0.5758 and 0.5683, respectively, both of which higher than that of Blending.

**Table 2.** Prediction accuracy of three methods on different simulated sets.

Trait	Blending	CAssBlending	PPssBlending
h0.2_q200	0.1733	0.1705	0.1641
h0.2_q2000	0.2351	0.2258	0.2409
h0.2_q5000	0.3663	0.3698	0.3624
h0.5_q200	0.5669	0.5683	0.5758
h0.5_q2000	0.4369	0.4329	0.4406
h0.5_q5000	0.4512	0.4518	0.4575
h0.8_q200	0.7870	0.7868	0.7892
h0.8_q2000	0.7865	0.7863	0.7859
h0.8_q5000	0.7566	0.7568	0.7568

*Notes:* h0.2_q200 represents a simulated dataset with the heritability of 0.2 and QTN number of 200; other representations are similar.

### 3.3 Prediction performance of different methods on real datasets

**Figure 4.**
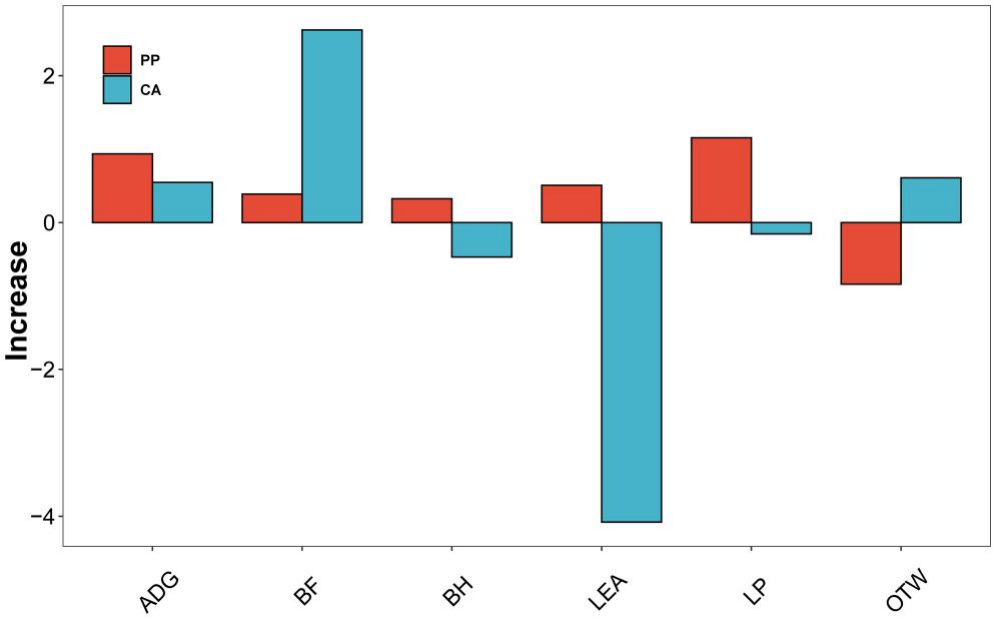
Increase in prediction accuracy of ssBlending, compared with Blending, for six pig traits.

In pig data, the prediction accuracy of blending for 6 traits (ADG, BF, LEA, OTW, BH, and LP) was 0.5280, 0.2026, 0.1308, 0.3164, 0.3557, and 0.27670, respectively, while that of PPssBlending was 0.5373, 0.2064, 0.1359, 0.3080, 0.3589, and 0.2883, and that of CAssBlending was 0.5334, 0.2288, 0.0900, 0.3225, 0.3510, and 0.2752, respectively (Table 3). Compared with blending, our ssBlending method exhibited an increase of 0.3242%–2.6236% in prediction accuracy for 6 traits (Fig. 4).

**Table 3.** Prediction accuracy of three methods for six different traits on real pig data.

Trait	Blending	CAssBlending	PPssBlending
ADG	0.5280	0.5334	0.5373
BF	0.2026	0.2288	0.2064
BH	0.3557	0.3510	0.3589
LEA	0.1308	0.0900	0.1359
LP	0.2767	0.2752	0.2883
OTW	0.3164	0.3225	0.3080

*Notes:* ADG, average daily weight gain; BF, backfat thickness; BH, body height; OTW, off test weight; LEA, loin eye area; LP, lean percentage.

**Figure 5.**
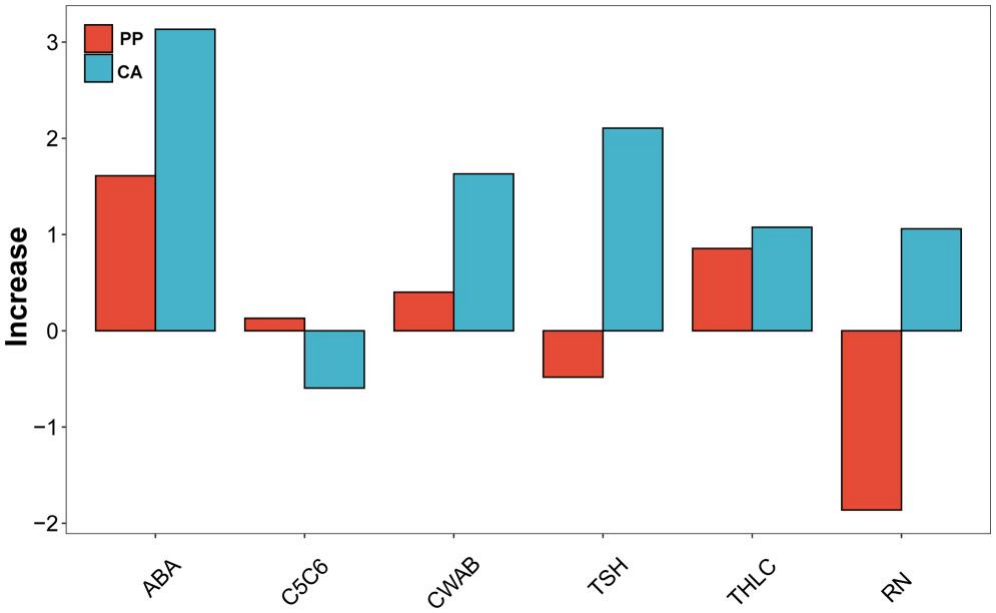
Increase in prediction accuracy of ssBlending, compared with Blending, for six loblolly pine traits.

The genetic structure of plants differs significantly from that of animals; therefore, we assessed this approach on six traits (RT, ABA, THLC, CWAB, TSH, and C5C6) of loblolly pine. Compared with the blending, the ssBlending exhibited a maximum increase in prediction accuracy of 3.1324% (Fig. 5). The average prediction accuracy of the blending for the six traits was approximately 0.2991, ranging from 0.1448 to 0.4306, while that of CAssBlending and PPssBlending was 0.3002 and 0.3131, respectively (Table 4), further confirming a higher stability and accuracy of our proposed new method, relative to conventional blending.

**Table 4.** Prediction accuracy of three methods for different traits on loblolly pine data.

Trait	Blending	CAssBlending	PPssBlending
ABA	0.4306	0.4619	0.4467
C5C6	0.1495	0.1435	0.1508
CWAB	0.4244	0.4407	0.4285
TSH	0.3188	0.3398	0.3140
THLC	0.3265	0.3373	0.3350
RN	0.1448	0.1554	0.1261

*Notes:* RN, root number; ABA, average branch angle; THLC, total height to the base of the live crown; CWAB, crown width across the planting beds; TSH, total stem height; C5C6: 5- and 6-carbon suger content.

**Figure 6.**
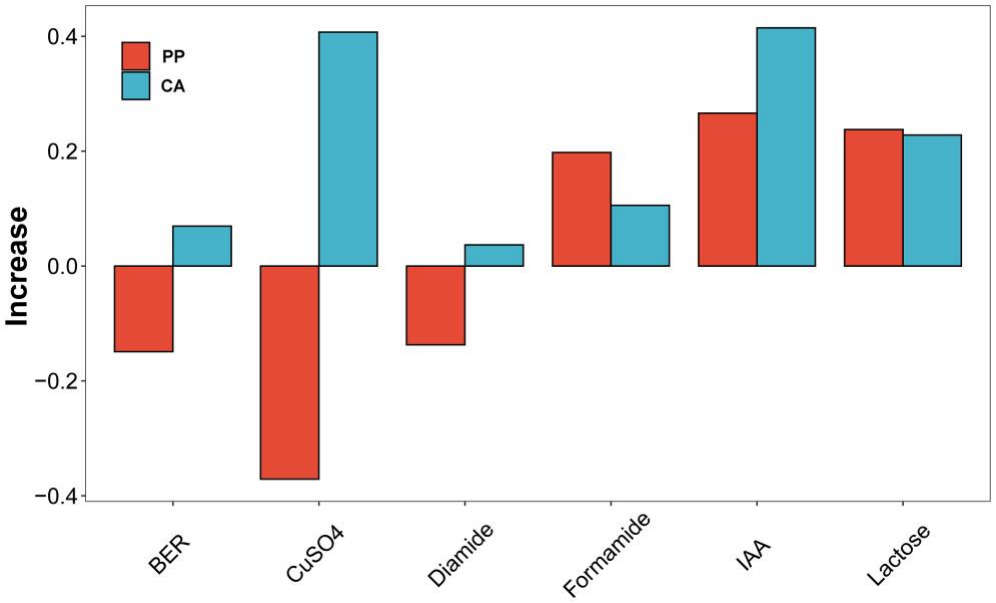
Increase in prediction accuracy of ssBlending, compared with blending, for six yeast traits.

Further, we performed validation on microbial data, using six yeast traits. The results showed that the prediction accuracy of ssBlending surpassed that of Blending (Table 5). For formamide, IAA, and lactose traits, the ssBlending exhibited an increase in prediction accuracy of 0.1056%–0.4146%, compared with blending (Fig. 6).

**Table 5.** Prediction accuracy of three methods for six different traits on yeast data.

Trait	Blending	CAssBlending	PPssBlending
BER	0.7608	0.7615	0.7593
CuSO_4_	0.7369	0.7410	0.7332
Diamide	0.7553	0.7557	0.7539
Formamide	0.6053	0.6063	0.6072
IAA	0.6298	0.6340	0.6325
Lactose	0.5979	0.6002	0.6003

*Notes:* CuSO_4_, copper sulfate; BER, berbamine; IAA, indol acetic acid.

In addition, we found in real data that when the distribution of the phenotypic values of the analyzed traits is more in line with the normal distribution, the PP strategy is usually better than the CA strategy; otherwise, the CA strategy is selected. The LP skewness of pigs is 0.0732, which is small (Supplementary Fig. S1), so the PP strategy is better than the CA strategy; TSH and RN of loblolly pine have larger skewnesses compared to other traits. In this case, the PP strategy is not as effective as the original Blending method. The skewness of C5C6 is smaller and closer to the normal distribution, so the PP method is worse than the CA method; the larger the skewness of traits such as ABA, CWAB and THLC, the more reasonable it is to choose the CA strategy (Supplementary Fig. S2). In the yeast data, the phenotypes of the five traits CuSO4, diamide, BER, formamide and IAA show large normal skewnesses. In this case, the PP method has no obvious advantage over the CA method (Supplementary Fig. S3).

Taken together, for different traits across different species (animal, plant, and microbe), our method shows certain advantages in various data with multiple different genetic structures.

## 4 Discussion

Stratified sampling is an effective method to accurately reflect overall characteristics of samples (Singh *et al.* 1996). When we apply stratified sampling for samples selection, the group is divided into smaller sub-groups, which are known as strata. By dividing the population into smaller groups or strata based on shared attributes, stratified sampling ensures the representativeness of demographic characteristics. And the studies performed for various learning tasks and many data sets have shown stratified sampling superiority with respect to random sampling (Cervellera and Macciò 2018). The method of selecting data stratification warrants careful consideration. Kim *et al* utilized K-means clustering and decision tree induction as hierarchical rules generated through data mining technology to segment and classify data, significantly enhancing the percentage of variance interpretation (Kim *et al.* 2013). Jing *et al.* (2015) proposed clustering high-dimensional data into several feature layers and employing stratified sampling to generate a component data set through random sampling within the feature layers. And their experimental results demonstrate that the clustering outcomes of stratified sampling outperform those of random sampling. Following these inspirations, we introduce a new variant of blending algorithm, namely, ssBlending, which incorporates stratified sampling strategy. In addition, we employed clustering analysis of genotypic data alongside the probability distribution of phenotypes to stratify the samples. Subsequently, we integrated these stratification strategies into the blending framework, which corresponded to clustering analysis ssBending and phenotypic probability ssBlending, respectively.

In order to reveal the role of stratified sampling in enhancing the prediction performance of the Blending algorithm, we conducted a comparative analysis between the ssBlending and Blending methods. Our results consistently revealed that ssBlending exhibited superior predictive performance, compared with Blending. Unlike blending relying on simple random sampling, ssBlending utilizes stratified sampling to ensure comprehensive coverage of features in the training set, thus increasing prediction accuracy. When the training set adequately represents the entire dataset, the prediction results will be reliable. The influence of four sampling methods, namely random sampling of training set and test set, systematic sampling, stratified random sampling, and stratified system sampling in machine learning is explored by Muhammed Kurs *et al.* It is demonstrated that the performance of the two stratified sampling methods outperforms others (Uçar *et al.* 2020). This underscores the importance of a well-structured and diversified training dataset for attaining desirable prediction performance in Blending algorithms. Overall, our study highlights the profound impact of implementing stratified sampling on the enhancement of the predictive performance of the ssBlending algorithm.

The superior prediction performance of ssBlending compared to conventional blending primarily stems from its stratified sampling strategy. Stratified sampling is advantageous in selecting sample sets more representative of the original population, compared to random sampling. By ensuring proper representativeness for each group within the population, stratified sampling enhances the accuracy of estimating model parameters, particularly when the population is divided into homogeneous subgroups. In general, stratified sampling is superior to simple random sampling when a robust machine learning model is trained on the training set of the population, especially when the population is heterogeneous or when the sample size is limited. However, simple random sampling is more advantageous when the population is homogeneous or lacks clear subgroup division.

The optimization of the data structure of the training set by stratified sampling can enhance the prediction accuracy of the ssBlending algorithm. Maximizing the inter-stratum phenotypic variance captured by the training set is critical for improving the prediction accuracy of the models. The comparison of ssBlending and Blending revealed that the prediction performance of ssBlending is better than that of the conventional Blending. Moreover, in machine learning, the selected parameters affect overall model performance, even for the same learner, the prediction results vary with parameters set (Petrak 2000, Thornton *et al.* 2013). Hence, we optimized parameter settings in this study thought training sets, and applied the BestH obtained in training to the test set for the prediction of unknown phenotypes. The objective of this methodology is to efficiently identify the parameter values that optimally align with the characteristics of the specified dataset, thereby enhancing the accuracy of ensemble learning. Our study also confirmed this optimization process also provides more effective prediction performance for ssBlending.

## 5 Conclusion

This study addresses a drawback of the blending algorithm, namely, the inaccuracy in prediction performance caused by its random sampling on training sets. We propose stratified sampling approach for training sets including clustering analysis strategy and phenotypic probability strategy. By replacing the simple random sampling with these two stratified sampling strategies, we introduce the ssBlending algorithm, which combines the advantages of heterogeneous ensemble learning algorithm and stratified sampling. Our data across different species reveal that ssBlending exhibits superior prediction performance, compared with the conventional blending algorithm, highlighting the potential of ssBlending in genome selection applications. By reducing the instability and inaccuracy of the blending algorithm via stratified sampling, the ssBlending provides an improved approach with high prediction accuracy for genome selection tasks. Notably, our proposed ssBlending method is not limited to genomic prediction, and the more applications of this method in other fields remain to be further investigated.

## Supplentary data

Supplementary data are available at *Bioinformatics Advances* online.

## Supplementary Material

vbaf002_Supplementary_Data

## Data Availability

The data underlying this article are available in the article and in its online supplementary material (https://figshare.com/s/23122a18dc8a35f12ff6).
