# Supplementary material for: A new ensemble learning method stratified sampling blending optimizes conventional blending and improves prediction performance
Source: Bioinform Adv. 2025 Feb 22;5(1):vbaf002. doi: 10.1093/bioadv/vbaf002 (PMC11908643; doi:10.1093/bioadv/vbaf002)
Supplement: vbaf002_Supplementary_Data [file vbaf002_supplementary_data.zip › 2cad7_ssblending_Supplementary.docx]

**A new ensemble learning method stratified sampling Blending (ssBlending) optimizes conventional blending and improves prediction performance**

Na Miao et al

Supplementary Information


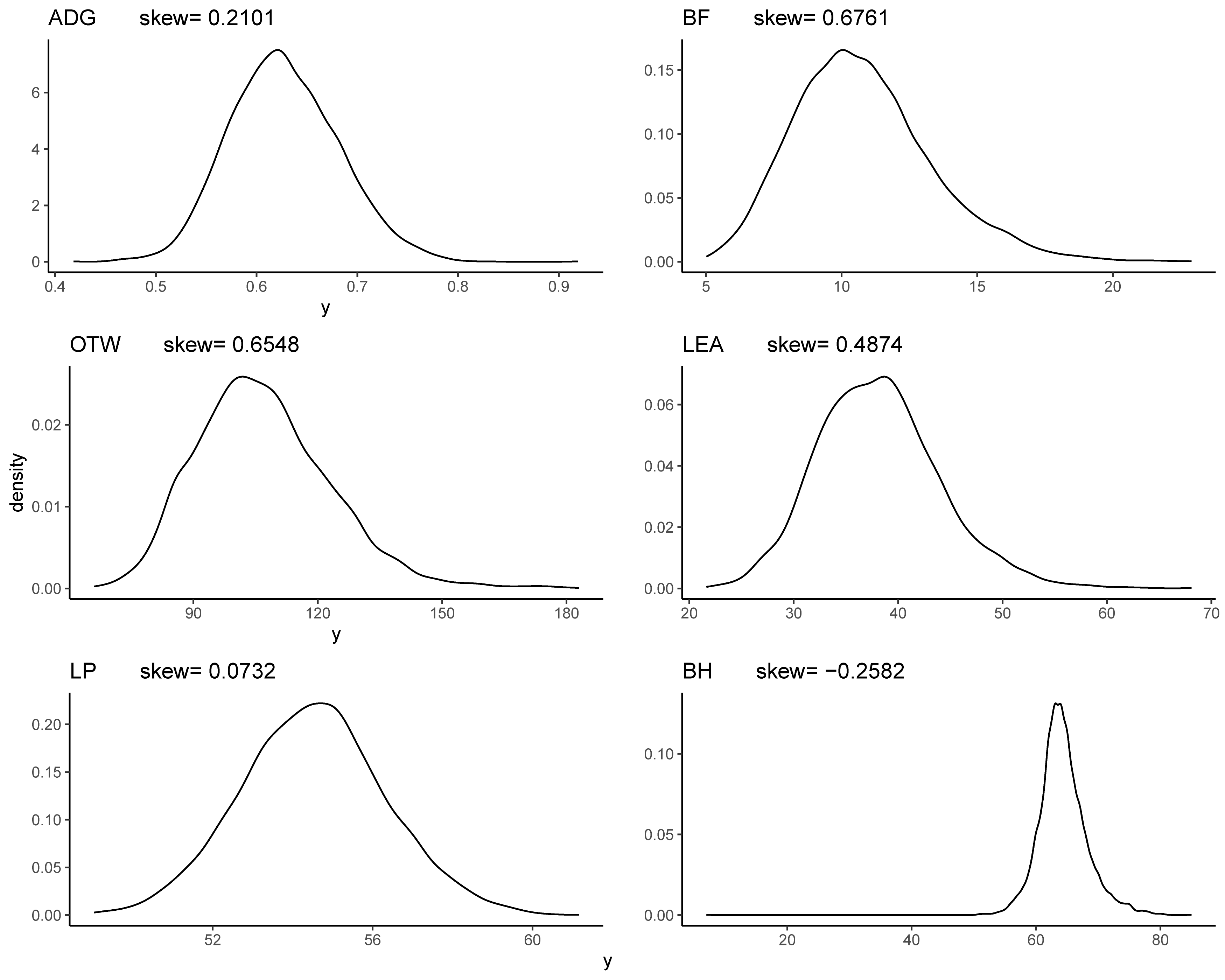


Supplemently Fig. 1 Phenotypic distribution of six traits in pig


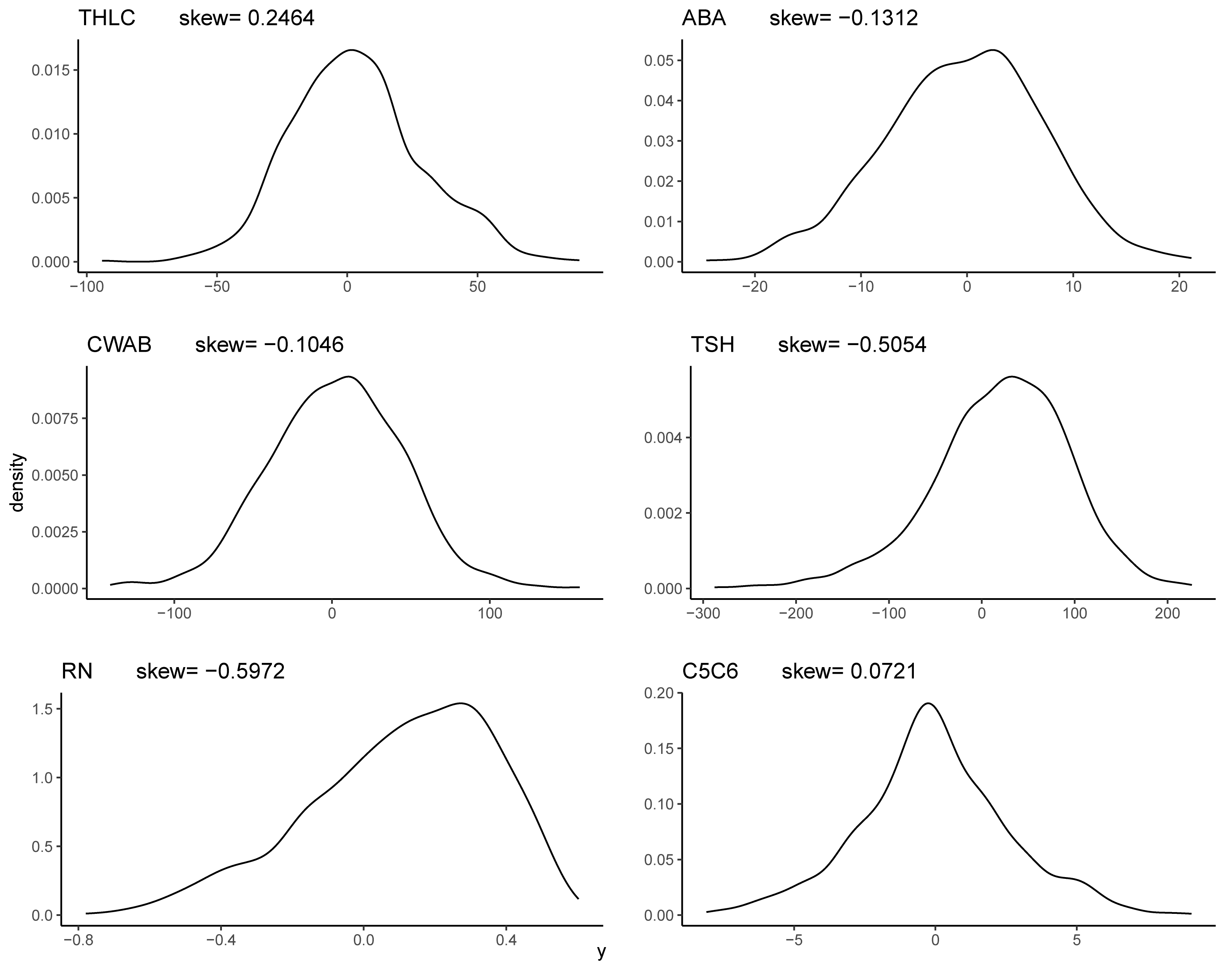


Supplemently Fig. 2 Phenotypic distribution of six traits in loblolly pine


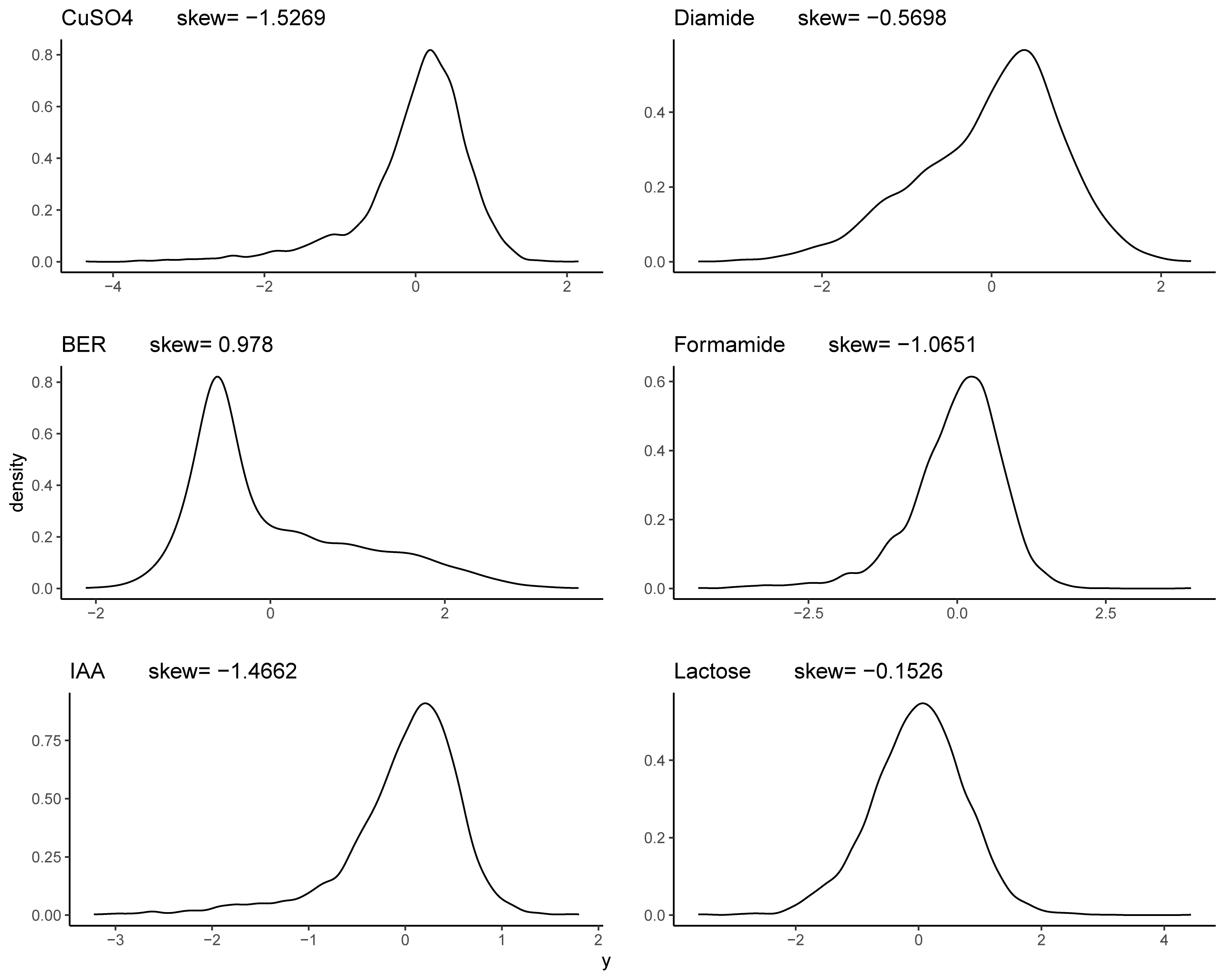


Supplemently Fig. 3 Phenotypic distribution of six traits in yeast

Supplemently Table. 1 Total time for 5-fold cross validation of Blending and ssBlending

| Data | Trait | Blending | CAssBlending | PPssBlending |
| --- | --- | --- | --- | --- |
| Simualte | h0.2_q200 | 10.1963 | 9.2715 | 9.9989 |
|  | h0.2_q2000 | 9.1505 | 10.4453 | 8.9250 |
|  | h0.2_q5000 | 10.6608 | 9.2028 | 9.9061 |
|  | h0.5_q200 | 9.3006 | 11.4517 | 10.4752 |
|  | h0.5_q2000 | 12.5499 | 10.7295 | 11.6047 |
|  | h0.5_q5000 | 11.6989 | 11.3470 | 12.0432 |
|  | h0.8_q200 | 11.8929 | 11.4826 | 13.3783 |
|  | h0.8_q2000 | 14.4004 | 12.7622 | 14.6164 |
|  | h0.8_q5000 | 16.8575 | 15.7625 | 15.8517 |
| Pig | ADG | 26.9728 | 26.4141 | 27.7213 |
|  | BF | 30.4553 | 28.9696 | 27.7812 |
|  | BH | 37.1345 | 34.8086 | 26.9397 |
|  | LEA | 24.7688 | 28.1009 | 22.9623 |
|  | LP | 24.8608 | 35.5978 | 34.3780 |
|  | OTW | 31.2547 | 31.1920 | 30.5921 |
| Loblolly pine | ABA | 0.6581 | 0.5954 | 0.6370 |
|  | C5C6 | 0.4781 | 0.4154 | 0.5032 |
|  | CWAB | 0.6438 | 0.5493 | 0.5868 |
|  | TSH | 0.5383 | 0.4633 | 0.5051 |
|  | THLC | 0.5543 | 0.5282 | 0.5272 |
|  | RN | 0.4726 | 0.4631 | 0.4773 |
| Yeast | CuSO_4_ | 29.0564 | 25.7166 | 22.0435 |
|  | BER | 23.4620 | 25.4646 | 26.3330 |
|  | Diamide | 30.4016 | 30.4990 | 33.2578 |
|  | Formamide | 18.7829 | 15.6687 | 15.6856 |
|  | IAA | 18.5117 | 14.3414 | 16.8967 |
|  | Lactose | 17.5178 | 17.1029 | 17.1702 |

Notes:Time unit is measured in hours
